# Modulation of TRPA1 channel activity by Cdk5 in sensory neurons

**DOI:** 10.1080/19336950.2018.1424282

**Published:** 2018-01-08

**Authors:** Michael A. Sulak, Monica Ghosh, Pritam Sinharoy, Spencer R. Andrei, Derek S. Damron

**Affiliations:** a Department of Human Genetics, University of Chicago, Chicago, IL, USA; b Department of Biological Sciences, Kent State University, Kent, OH, USA; c Department of Anesthesia, Perioperative and Pain Medicine, Stanford School of Medicine, Stanford, CA, USA; d Department of Medicine, Vanderbilt University Medical Center, Nashville, TN, USA

**Keywords:** Ca^2+^ signaling, Cdk5, DRG neurons, ion channel, TRPA1

## Abstract

Transient receptor potential cation channel, subfamily A, member 1 (TRPA1), is activated by a broad range of noxious stimuli. Cdk5, a member of the Cdk family, has recently been identified as a modulator of pain signaling pathways. In the current study, we investigated the extent to which Cdk5 modulates TRPA1 activity. Cdk5 inhibition was found to attenuate TRPA1 response to agonist in mouse DRG sensory neurons. Additionally, the presence of active Cdk5 was associated with increased TRPA1 phosphorylation in transfected HEK293 cells that was roscovitine-sensitive and absent in the mouse mutant S449A full-length channel. Immunopurified Cdk5 was observed to phosphorylate human TRPA1 peptide substrate at S448A *in vitro*. Our results point to a role for Cdk5 in modulating TRPA1 activity.

## 1. Introduction

TRPA1 is a polymodal ion channel expressed in a subset of small-diameter, capsaicin-sensitive, nociceptive neurons of the trigeminal and dorsal root ganglia, the activation of which produces pain and neurogenic inflammation [1]. The sole member of the TRPA subfamily expressed in mammals, TRPA1 has recently emerged as a key nociceptive mediator, responsive to a multitude of stimuli, including the pungent molecules, allyl isothiocyanate (AITC) [2] and propofol (an intravenous anesthetic) [3]. While this non-selective cation channel has received increasing experimental scrutiny of late, most studies have focused on identifying TRPA1 ligands, with receptor regulation receiving less attention.

Cdk5, a member of the Cdk family which is principally active in post-mitotic neurons, plays multiple vital roles in the nervous system [4], but its recent emergence as a potential ‘executive’ or ‘master’ regulator of multiple pain signaling pathways [5,6] is what's of greatest relevance in the context of the present investigation. While not regulated by cyclins, Cdk5 activity is dependent upon binding to one of its two regulatory proteins [7], p35 or p39.

p35 knockout mice and Cdk5 conditional (neurons only) knockout mice are less sensitive to noxious thermal stimuli than wild-type mice, while mice over-expressing p35 are hyperalgesic [8]. Cdk5 inhibition reduces capsaicin-induced Ca^2+^ influx in cultured DRG neurons [9] and attenuates the formalin-induced flinch response in rats [10]. Separate studies have demonstrated the primacy of TRPA1 as the mediator of formalin-induced pain [11–13]. Considering these facts, the possibility that Cdk5 might act as a regulator of TRPA1 signaling emerges. This investigation tests the hypothesis that Cdk5 modulates the activity TRPA1, a nociceptive mediator of increasingly recognized importance.

## 2. Materials and methods

### 2.1. Animal models

Male C57BL/6 mice, 12 weeks old, were utilized in these studies. All animals were housed at Kent State University in an animal facility accredited by the Association for Assessment and Accreditation of Laboratory Animal Care.

### 2.2. DRG neuron isolation and culture

DRG were dissected from the lumbar spinal cord of mice, incubated with collagenase (Type IV, 0.15%) at 37°C for 50 min, then dissociated by gentle trituration. Neurons were plated in 6-well dishes onto coverslips pre-coated with poly-D-lysine/laminin and cultured in a humidified atmosphere at 37°C and 5% CO_2_ in Ham’ s F-12K medium supplemented with 10% fetal bovine serum (FBS), 100 ng/ml nerve growth factor and antibiotics. Proliferation of fibroblasts and Schwann cells was inhibited by addition of cytosine arabinoside (5–10 μM) to the medium. Studies were performed 24–48 hours after isolation.

### 2.3. HEK293 cell culture

HEK293 cells were obtained from the American Type Culture Collection and grown in 10 cm dishes in Dulbecco's Modified Eagle's Medium supplemented with 10% FBS and antibiotics (penicillin (100 U/ml), streptomycin (100 mg/ml)) in a humidified atmosphere at 37° Celsius and 5% CO_2_.

### 2.4. Intracellular Ca^2+^ measurements

Intracellular free Ca^2+^ concentration ([Ca^2+^]_i_) measurements were performed on DRG neurons and HEK293 cells using a microscope based-fluorescence imaging system (Easy Ratio Pro, Horiba Scientific, Edison, NJ.), as described previously [14].

### 2.5. Plasmid transfection

Plasmids were transfected as previously described [15] using Lipofectamine 2000 (Invitrogen) according to the manufacturer's recommendations. Myc-tagged mouse cDNA for TRPA1 was the kind gift of Armen Akopian, Ph.D. (University of Texas Health Science Center at San Antonio). GFP-tagged Cdk5 and p25 were obtained from Addgene (Cambridge, MA.), through the courteous donation of Li-Huei Tsai, Ph.D. (Picower Institute, Cambridge, MA.). TRPV1 cDNA was kindly provided by David Julius (Ph.D., Professor, Dept. of Physiology, University of California at San Francisco, San Francisco, California) and was transfected into all HEK293 cells 24 hrs prior to any other transfection protocols.

### 2.6. Site-directed mutagenesis

Site-directed mutagenesis on mouse TRPA1 obtained from (Armen Akopian, Ph.D.) was performed by polymerase chain reaction (PCR) using Quickchange II – Site-directed mutagenesis kit (Agilent Technologies) according to manufacturer's protocol. S449A (mouse numbering) TRPA1 mutant was generated using S449A Forward primer: 5′ CAAAAGTAAAGATAAGAAGGCGCCCCTGCATTTTGCAG 3′ and Reverse primer: 5′ CTGCAAAATGCAGGGGCGCCTTCTTATCTTTACTTTTG 3′ (Integrated DNA Technologies). S449A mutation on TRPA1 was confirmed by DNA sequencing (Cleveland Clinic Genomics Core).

### 2.7. Cell and tissue lysis

500 µL of lysis buffer consisting of 150 mM NaCl, 50 mM Tris HCl (pH 7.4), 5 mM EDTA, 1% Triton X-100, 1 mM DTT, 1 mM PMSF, Halt Protease inhibitor cocktail (1:100, Pierce/Thermo), and PhosSTOP phosphatase inhibitor cocktail (1:9, Roche Applied Science) was added to confluent, 10 cm plates of HEK293 cells, which were then scraped and pipetted into 1.5 mL tubes. Cell lysis was completed by rotating tubes for 45 minutes at 4° Celsius, followed by centrifugation at 7,000x g for 20 minutes (4° Celsius). Supernatants were assayed for protein content via modified Lowry assay (Biorad). Rat Brain tissue was polytron-homogenized in lysis buffer (described above), mixed on a rotator for 45 minutes and centrifuged at 10,000x g for 20 minutes (all at 4° Celsius). Supernatant protein was quantified as described above.

### 2.8. Immunoprecipitation

HEK293 cell lysates were diluted in lysis buffer such that equal protein quantities (500–1500 µg) and equal volumes (500–600 µL) were loaded into experimental and control tubes. Samples were pre-cleared for 60 minutes in 40 µL pre-washed protein A/G agarose bead slurry (Santa Cruz Biotechnology), then incubated overnight with 7 µg anti-myc tag IgG (sc-40, Santa Cruz Biotechnology) at 4° Celsius on a rotator. The following day, samples were added to 70 µL pre-washed protein A/G agarose bead slurry and rotated for two hours at 4° Celsius. Sample tubes were centrifuged briefly and supernatants were removed. Beads were washed four times in lysis buffer and once in PBS. After the final wash, residual buffer was aspirated from the beads with a fine-gauge needle, Laemmli buffer was added to each tube, and samples were boiled for 10 minutes in preparation for electrophoretic separation. Rat brain lysate-derived, immunoprecipitated, Cdk5 served as the enzyme source for kinase assays, following the method of Pareek, et al [9].

### 2.9. Electrophoresis and western blotting

4–15% gradient polyacylamide gels (Biorad) were loaded with 35–40 µL bead supernatant per well and run for 1 hour at 140 volts. Proteins were electrophoretically transferred to nitrocellulose membranes (Biorad) for 1 hour at 85 volts. Membranes were blocked in 7% milk in TBST containing 50 mM NaF (TBSTF), washed 3–4x in TBSTF, then incubated overnight in anti-phosphoserine IgG (Sigma-Aldrich), diluted 1:1000 in TBSTF containing 5% bovine serum albumin. Membranes were washed 3–4x in TBSTF, then incubated for 1 hour with HRP-conjugated, goat anti-mouse secondary antibody (1:10,000, Santa Cruz Biotechnology), followed by 4 additional washes in TBSTF. Immunoreactive bands were visualized via enhanced chemiluminescence (Pierce/Thermo) and exposed to X-ray film. Band density was normalized to total TRPA1 after stripping membranes and reprobing with anti-myc tag IgG (sc-40, Santa Cruz Biotechnology). Signal intensity was quantified using ImageJ (NIH).

### 2.10. In vitro peptide kinase assay reactions

Cdk5 immunoprecipitates from rat brain were combined with TRPA1 peptides (either S448 wild-type or S448A mutant, each at 0.2 µM, Biomatik) and 5 µCi of [^32^P] ATP (MP Biomedicals), then incubated at 30° Celsius for 40 or 70 minutes (total volume of each reaction = 50 µL). Reactions were quenched with 70 µL 10% trichloroacetic acid. Samples were subsequently centrifuged for 5 minutes at 10,000x g, and 17 or 20 µL supernatant aliquots were spotted onto P81 phosphocellulose squares. Each reaction condition was performed in triplicate, and each replicate reaction was spotted onto phosphocellulose squares in triplicate (A total of nine squares per reaction condition). After air-drying in a fume hood, squares were individually washed in 6-well plates with 75 mM Phosphoric acid (5 washes, 15–20 minutes each). A final 15 minute wash in acetone was followed by air-drying. Dried squares were placed in scintillation vials containing biosafe scintillation fluid, (ScintiVerse™ BD Cocktail, Fisher Scientific) and quantified in a Beckman LS-6500 scintillation counter. For inhibitor kinase assays, PD-98059 or Roscovitine (each at a final concentration of 15 µM, LC Laboratories, Woburn, Massachusetts), or vehicle alone (DMSO, final dilution, 1:3333) were added to immunoprecipitates, prior to addition of peptides and [^32^P] ATP.

### 2.11. Peptides

S448 wild-type (human TRPA1 numbering) and S448A mutant peptides corresponding to the sequence surrounding residue serine 448 of Human TRPA1 were obtained from Biomatik (Wilmington, DE.) for *in vitro* kinase assays. The 13-mer peptide sequences used were KSKDKKSPLHFAA (S448 wild-type) and KSKDKKAPLHFAA (S448A mutant).

### 2.12. Chemicals

Unless noted otherwise, chemicals and reagents were obtained from Sigma-Aldrich. Propofol was obtained from the Cleveland Clinic pharmacy.

### 2.13. Statistical analysis

Experiments were replicated three or more times, as noted in the figure captions. Statistical evaluation was done with Sigmaplot software (Systat Software, Inc.). Significant differences between experimental groups were assessed by one-way ANOVA (more than two groups) or unpaired Student's *t*-test (two groups). ANOVA was followed by Student's *t*-tests with Bonferroni's post-hoc correction for multiple comparisons, where significance was set at *p* < 0.05. Summarized data are expressed as means ± SEM.

## 3. Results

### 3.1. Cdk5 inhibition attenuates TRPA1 agonist-induced increases in [Ca^2+^]_i_ in DRG neurons, but not in TRPA1-transfected HEK293 cells, which lack Cdk5 activity

**Figure 1.**
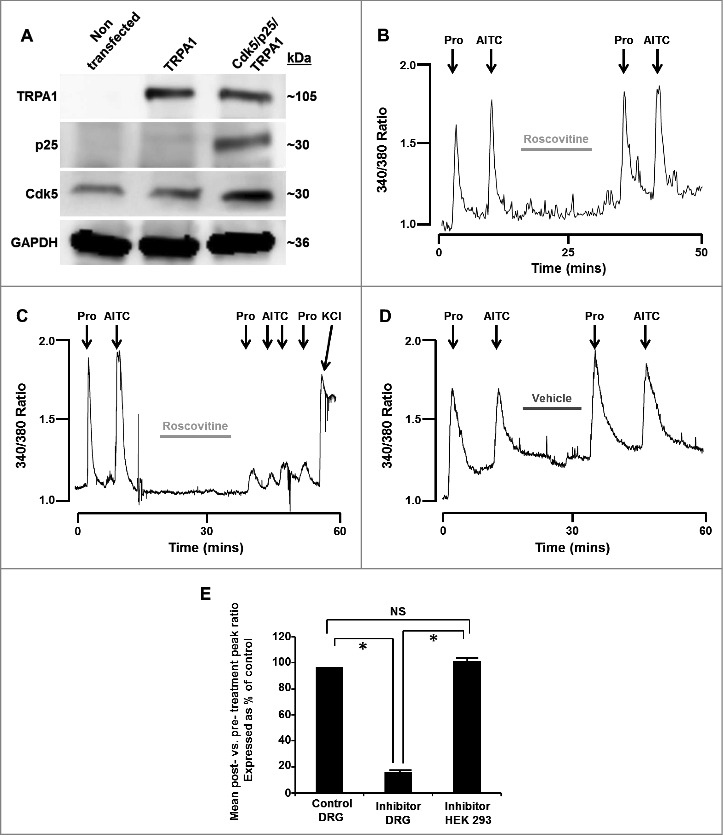
(A) Representative western blot images depicting the presence or absence of TRPA1, p25 or Cdk5 in HEK293 cells that were either non-transfected or transfected with TRPA1 only or Cdk5, p25 and TRPA1. GAPDH was probed as a loading control. (B) Representative trace depicting [Ca^2+^]_i_ transients induced by propofol and AITC (TRPA1 agonists) in the absence and presence of roscovitine (50 µM) in TRPA1/TRPV1-transfected HEK293 cells (which lack Cdk5 activity). Cells were exposed to propofol (Pro, 10 µM) and AITC (100 µM) for 60 seconds at the time points marked by arrows. (C) Representative trace depicting [Ca^2+^]_i_ transients induced by propofol and AITC in the absence and presence of roscovitine (50 µM) in DRG neurons. Cells were exposed to potassium chloride (KCl, 50 mM) for 30 seconds to demonstrate cell viability after exposure to roscovitine. (D) Representative control trace depicting [Ca^2+^]_i_ transients induced by propofol and AITC in the absence and presence of DMSO vehicle (0.1%) in DRG neurons. E) Summarized data for experiments represented in panels B-D expressed as % of control ± SEM. NS = Not significant. n = 8 separate experiments, *p* < 0.05.

Although cultured HEK293 express low levels Cdk5, they do not express the neuron specific activator p25 (Fig. 1A) and therefore Cdk5 cannot be constitutively active in cultured HEK293 cells [16–18]. Fura-2 loaded HEK293 cells transfected with TRPA1 and TRPV1 were first exposed to TRPA1 agonists (propofol (10 µM) and/or AITC (100 µM), to establish baseline response levels, and then underwent a 20 minute superfusion with HBSS containing the Cdk5 inhibitor, roscovitine (50 µM; Fig. 1B). A 2^nd^ exposure to TRPA1 agonists followed, testing TRPA1 responses in cells exposed to Cdk5 inhibition vs. controls. Roscovitine had no effect on propofol or AITC induced increases in [Ca^2+^]_i_. However, in isolated fura-2 loaded DRG sensory neurons undergoing the same protocol, roscovitine markedly attenuated propofol and AITC-induced increases in [Ca^2+^]_i_ (Fig. 1C). A marked increase in [Ca^2+^]_i_ was observed in response to KCl following roscovitine exposure indicating cell viability and an intact membrane potential. The DMSO vehicle for roscovitine was without effect on propofol or AITC induced increases in [Ca^2+^]_i_ (Fig. 1D).

Summarized data represent the ratio of peak heights post-exposure to vehicle or inhibitor compared to pre-exposure peak heights (Fig. 1E). Mean post:pre-exposure peak height ratios were 0.97 in control DRG neurons, 0.157 in inhibitor-exposed DRG neurons and 1.01 in inhibitor-exposed HEK293 cells. Inhibitor-exposed DRG ratios differ significantly from both control DRG ratios and inhibitor-exposed HEK293 cell ratios (*p* ≤ 0.01). Control DRG neuron ratios and inhibitor-exposed HEK293 cell ratios were not significantly different.

### 3.2. Inhibition of Cdk5 activity attenuates TRPA1 agonist-induced increases in [Ca^2+^]_i_ in DRG neurons in a reversible and dose-dependent manner

**Figure 2.**
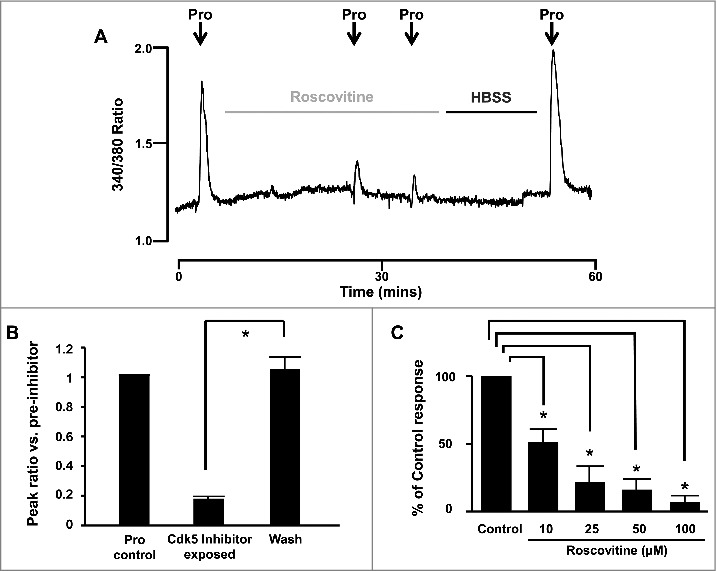
(A) Representative trace demonstrating the reversible attenuation of propofol-induced [Ca^2+^]_i_ transients effected by roscovitine (50 µM, incubation time indicated by light gray line) in mouse DRG neurons. (B) Summarized data for experiments represented in panel A expressed as peak ratio vs. pre-inhibitor ± SEM. n = 8 separate experiments, *p* < 0.05. Cells were exposed to propofol (Pro, 10 µM) for 60 seconds at the time points marked by arrows. (C) Summarized data for dose-dependence experiments. Error bars = SEM. n = 8 separate experiments, *p* < 0.05.

Fura-2-loaded DRG neurons were exposed to the TRPA1 agonist, propofol, to establish baseline response levels, followed by a 20 minute superfusion with HBSS containing the Cdk5 inhibitor, roscovitine (50 µM). A 2^nd^ exposure to propofol was performed to assess TRPA1 responses subsequent to Cdk5 inhibition. Following 20 minutes washout in HBSS, a final exposure to propofol demonstrated the reversible nature of the TRPA1 response attenuation. A representative trace is shown in Fig. 2A. Mean peak heights in inhibitor exposed cells were 16.5% of pre-exposure levels, while mean post-washout peak heights were at 99% of pre-exposure levels (Fig. 2B). The dose-dependent nature of the inhibition was established by following the same basic protocol, but varying the concentration of inhibitor (10–100 µM). Summarized data are given as the ratios of peak heights post-exposure vs. pre-exposure to inhibitor expressed as percentage of control (Fig. 2C).

### 3.3. In vitro phosphorylation of TRPA1 peptide at Serine 448 is blocked by the Cdk5 inhibitor, roscovitine, but not by the MAPK inhibitor, PD-98059

**Figure 3.**
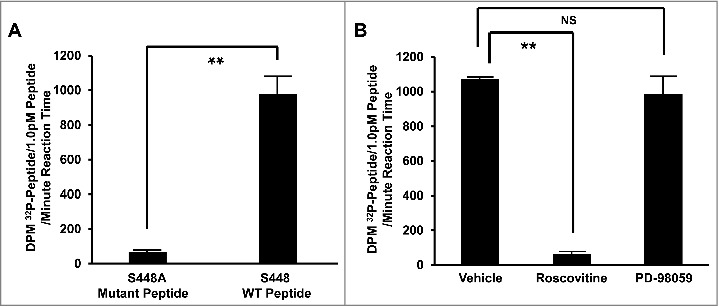
(A) Summarized data depicting differential Cdk5-mediated phosphorylation of mutant (S448A) and wild-type (S448 WT) TRPA1 peptide substrates. (B) Summarized data depicting the effect of Cdk5 inhibition (roscovitine, 50 µM), MAPK inhibition (PD-98059, 15 µM), or vehicle alone (DMSO, 0.03%), on phosphorylation of wild-type (S448 WT) peptide substrates. For both A and B, bars represent mean disintegrations per minute (DPM) ^32^P per picomole of peptide spotted onto P81 phosphocellulose squares per minute of *in-vitro* kinase assay reaction time ± SEM. n = 6, *p* ≤ 0.001. NS = Not significant.

To test the competence of Cdk5 to specifically phosphorylate TRPA1 residue S448 (human numbering), *in vitro* kinase assays were performed using both S448 wild-type and S448A mutant peptide substrates. Mean disintegrations per minute (DPMs) were 15.4-fold higher in wild-type peptide vs. S448A mutant peptide (Fig. 3A), indicating markedly lower phosphorylation of mutant peptide compared to wild-type.

In order to further confirm the Cdk5 specificity of S448 phosphorylation, *in vitro* kinase assays using S448 wild-type peptide substrates were repeated in the presence of either roscovitine (Cdk5 inhibitor), PD-98059 (MAPK inhibitor), or DMSO (vehicle). Cdk5 inhibition resulted in a marked (highly significant) reduction in phosphorylation compared to vehicle-treated control. MAPK inhibition with PD-98059 had no significant effect on phosphorylation compared to vehicle-treated control. Radioactive counts were 17.3-fold higher in vehicle vs. roscovitine-treated peptides, *p* < 0.001, while PD-98059-treated peptides did not differ significantly from vehicle-treated (Fig. 3B).

### 3.4. The presence of active Cdk5 is associated with increased TRPA1 phosphorylation in transfected HEK293 cells, as demonstrated by phosphoserine western blot

**Figure 4.**
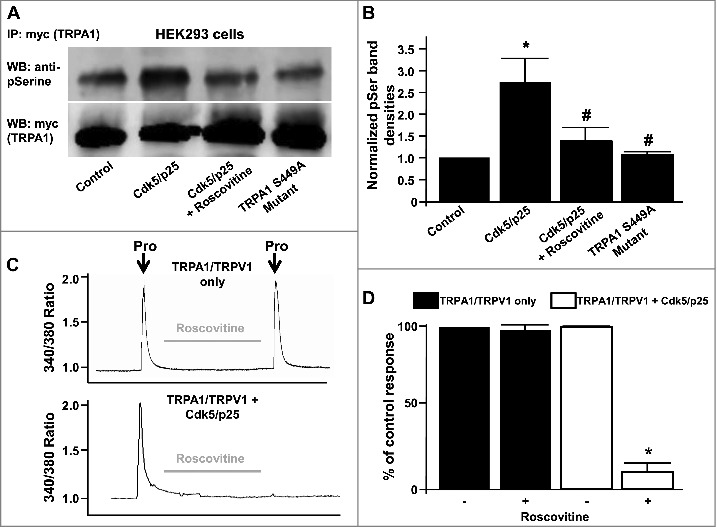
(A) Representative western blot depicting phosphoserine immunoreactivity in HEK293 lysates from myc-tagged TRPA1 (myc-TRPA1) expressing cells co-transfected with either control RFP vector, Cdk5/p25 (in the presence or absence of roscovitine) or TRPA1 S449A mutant cDNAs. Lower blot shows myc-TRPA1 immunoreactivity in the same lanes after stripping and reprobing. (B) Summarized data for panel A, illustrating normalized TRPA1 phosphoserine immunoreactivities ± SEM. n = 6, *p* < 0.05. (C) Representative traces depicting effect of roscovitine on propofol-induced (Pro, 10 µM) transient increase in [Ca^2+^]_i_ in HEK293 cells expressing TRPA1/TRPV1 only (top) or TRPA1/TRPV1 and Cdk5/p25 (bottom). (D) Summarized data for panel C expressed as percent of the control response normalized to 100% ± SEM. n = 8 separate experiments, *p* < 0.05.

HEK293 cells were either triply-transfected with myc-tagged TRPA1/Cdk5-GFP/p25, TRPA1-S449A/Cdk5/p25 (experimental), or doubly-transfected with myc-tagged TRPA1/RFP empty vector (control) 24 hrs after transfection with TRPV1. Myc-tagged mouse TRPA1 was immunoprecipitated from transfected HEK293 cells with anti-myc IgG, and subjected to western blot with anti-phosphoserine IgG. TRPA1 phosphoserine immunoreactivity was quantified and normalized to total TRPA1 after stripping and reprobing with anti-myc IgG, to correct for differences in transfection efficiency and loading. Baseline levels of TRPA1 phosphoserine immunoreactivity were observed in the control, non-Cdk5/p25-transfected state (Fig. 4A). Moreover, a marked reduction in pSer phosphorylation of TRPA1 was observed in transfected HEK293 cells exposed to roscovitine for 20 min prior to the assay. Finally, no significant increase in pSer phosphorylation of TRPA1 was observed in HEK293 cells transfected with the TRPA1 S449A mutation of the full length protein compared to control (Fig. 4A). Cdk5/p25-transfected cells showed a marked increase (2.8 fold +/− 0.49) in TRPA1 phosphorylation compared to control and this effect was markedly attenuated following treatment with roscovitine or mutation of S449A in the full length channel protein (Fig. 4B). In native HEK293 cells lacking p25 that were transfected with TRPA1/TRPV1, roscovitine failed to have any effect on propofol-induced increases in [Ca^2+^]_i_ (Fig. 4, panel C, top). In contrast, when HEK293 cells were also transfected with Cdk5/p25, roscovitine virtually abolished the propofol-induced increase in [Ca^2+^]_i_ (Fig. 4, panel C, bottom). The summarized data for Fig. 4, panel C are depicted in Fig. 4, panel D.

## 4. Discussion

At 1100+ amino acids per subunit, TRPA1 offers abundant scope for rich and varied regulation of channel function. Despite widespread recognition of the receptor's significance however, the mechanisms regulating TRPA1 activity remain poorly defined. As a previous *in-vivo* investigation indicated that Cdk5 inhibition attenuates the formalin-induced flinch response in rats [10], and other, subsequent investigations demonstrated the primacy of TRPA1 as a mediator of formalin-induced pain [11–13], experiments were designed to test the hypothesis that Cdk5 modulates the activity of TRPA1 in both DRG sensory neurons and in TRPA1/TRPV1 co-transfected HEK293 cells. Only one previous study has implicated Cdk5 as a modulator of TRPA1 activity in transfected HEK 293 cells through a phosphorylation of TRPA1 at T673^19^. In that study, they concluded that T673 which is located outside the ankyrin-repeat (AR) domain is the only possible target of Cdk5. The results of our current investigation provide additional physiological and biochemical evidence beyond that of Hynkova et al [19]. for Cdk5 modulation of TRPA1 activity in both DRG sensory neurons and in transfected HEK293 cells. Moreover, we identify S448 located in the AR-12 domain as a target of Cdk5. These novel data are consistent with a regulatory mechanism involving direct phosphorylation of the receptor at S448 by Cdk5.

### 4.1. Expression patterns and mechanistic regulatory features of TRPA1 activity in sensory neurons; Role of transient receptor potential of the vanilloid subtype 1 (TRPV1)

TRPA1 expression was first identified as a cold-gated and putative mechanosensitive ion channel [20]. It is predominantly expressed in a subset of sensory neurons located in the dorsal root and trigeminal ganglia where it is primarily co-expressed with TRPV1 (<3% express TRPA1 only) as well as calcitonin gene related peptide and substance P. Physiological studies have since indicated that TRPA1 channels play an important role in in the development of thermal and/or mechanical hyperalgesia in certain inflammatory and nerve injury pain models [1,21–23]. Moreover, many TRPA1 channel-mediated responses appear to be regulated by TRPV1 [24–26] and the features of neuronal TRPA1 are not duplicated in cells expressing only TRPA1 and, instead, can be restored only when TRPA1 and TRPV1 are coexpressed [27]. Therefore, when studying the physiological, biochemical and biophysical regulation of TRPA1 channels, it is essential that both TRPV1 and TRPA1 be co-expressed when using heterologous expression systems. This approach was not used in the recent study by Hynkova et al., 2016. It's interesting to note that several recent studies have demonstrated Cdk5-mediated regulation of TRPV1 activity and modulation of pain related behavior and sensitivity [6,28,29].

### 4.2. Cdk5 inhibition attenuates TRPA1 agonist-induced increases in [Ca^2+^]_i_ in DRG neurons, but not in TRPA1/TRPV1-transfected HEK293 cells, which lack Cdk5 activity

Incubation with the Cdk5 inhibitor, roscovitine, resulted in a significant attenuation of the rise in [Ca^2+^]_i_ induced by TRPA1 agonists, while vehicle-treated DRG neurons failed to exhibit reduction in [Ca^2+^]_i_ signal upon re-treatment with TRPA1 agonists. As an additional control, inhibition experiments were repeated using TRPA1-transfected HEK293 cells, rather than DRG neurons, as the model system. Cdk5 inhibition is expected to be without effect in this cell line, due to a lack of significant Cdk5 activity [30,31]. Results indicate that Cdk5 inhibition has no influence on rise in [Ca^2+^]_i_ in response to TRPA1 agonists in transfected HEK293 cells, providing evidence for the target-specific action of the Cdk5 inhibitor, and confirming that attenuation of rise in [Ca^2+^]_i_ is not due to direct inhibitor interaction with TRPA1 itself.

An intriguing aspect of these results concerns the context-dependence of the requirement for Cdk5 activity for TRPA1 function. When endogenously expressed in the ‘native’ nociceptor environment of DRG neurons (with the full coterie of regulatory and binding partners present), TRPA1 response to agonist demonstrates a dependence upon Cdk5 activity. When transfected into the ‘artificial,’ non-nociceptor context of HEK293 cells however, the presumably more ‘naked’ TRPA1 receptor (lacking in regulatory factors and binding partners present in nociceptive neurons) responds robustly to agonist stimulation, in a manner independent from Cdk5 activity. This is most likely due to the fact that HEK293 cells do not express the neuron specific p25 regulatory factor required for Cdk5 activation [16,17,30]. Moreover, these results would seem to imply that a tonic inhibition of TRPA1 activity is present in DRG neurons (but not in HEK293 cells), and that this tonic inhibition is relieved by Cdk5 activity. Determining the identity of regulatory factors involved in this nociceptor-specific, tonic inhibition of TRPA1 function stands as a goal for future studies.

That a more layered and intricate regulatory machinery would have evolved to control and modulate TRPA1 nocisensor function within the highly-specialized environment of the nociceptive sensory neuron appears eminently logical. The placement of Cdk5 within such a regulatory framework is also far from surprising – as a factor principally active in post-mitotic neurons, which has previously been demonstrated to play key roles in multiple aspects of neuronal function – including pain signaling [5,8,32]. Cdk5 seems an apt factor to play a modulatory role in TRPA1-related nociception.

### 4.3. Inhibition of Cdk5 activity attenuates TRPA1 agonist-induced increases in [Ca^2+^]_i_ in DRG neurons in a reversible and dose-dependent manner

Kinase phosphorylation of substrates is generally a short-term modification unless actively maintained, due to ubiquitous protein phosphatase activity. Thus, incubation with kinase inhibitors leads to rapid de-phosphorylation of kinase substrates – and just as readily, washout of inhibitor, by returning the kinase to an active state, allows for rapid re-phosphorylation of substrates. Therefore, TRPA1 modulation by Cdk5 activity, whether by direct or indirect interaction, would be expected to be a reversible on/off process. Our results demonstrate that TRPA1 responses exhibit the anticipated reversibility under the influence of Cdk5 inhibition/washout, congruent with channel modulation by kinase action. Additionally, Cdk5 inhibition with roscovitine exhibited a dose-dependent effect on TRPA1 responses, as expected for a biological response to enzyme inhibition.

### 4.4. In vitro phosphorylation of TRPA1 peptide at Serine 448 (S448) is blocked by the Cdk5 inhibitor, roscovitine, but not by the MAPK inhibitor, PD-98059

In the interest of locating potential Cdk5 phosphorylation sites on TRPA1, bioinformatics searches were performed using the publically-available NetPhosK [33] and Scansite [34] programs. The TRPA1 residue returning the highest score as a potential Cdk5 consensus site was S448 (human numbering). To investigate whether S448 could act as a Cdk5 substrate, *in vitro* Cdk5 kinase assays were performed using wild-type S448 and S448A mutant peptide substrates. Our findings indicate robust Cdk5 phosphorylation of the wild-type S448 peptide, while phosphorylation levels for the S448A mutant peptide were close to background levels – clearly demonstrating that Serine 448 can act as a Cdk5 substrate *in vitro*. These results were further bolstered by Cdk5 kinase assays performed in the presence of either PD-98059 (MAPK inhibitor) or roscovitine (Cdk5 inhibitor). Cdk5 specificity of the observed phosphorylation was reliably confirmed in these assays, as Cdk5 inhibition significantly reduced TRPA1 peptide phosphorylation, while MAPK inhibition had no significant effect on peptide phosphorylation relative to uninhibited control.

### 4.5. The presence of active Cdk5 is associated with increased TRPA1 phosphorylation in transfected HEK293 cells, as demonstrated by phosphoserine western blot

In light of the altered TRPA1 physiological responses observed with Cdk5 inhibition in DRG neurons, attempting to ascertain the mechanism(s) by which Cdk5 influences TRPA1 activation is a logical next goal. Phosphorylation of channel residues as a means of regulation has been previously reported in multiple other members of the TRP superfamily, including TRPV1, TRPV4 and TRPV6 [28–30,35–37], and recently TRPA1^19^. To investigate the effect of Cdk5 activity on global serine phosphorylation of the TRPA1 ion channel, phosphoserine immunoreactivity was probed via western blot of TRPA1 immunoprecipitated from TRPA1/Cdk5/p25 co-transfected (experimental) and TRPA1/RFP empty vector co-transfected (control) HEK293 cells. Data from the present study indicate that the addition of Cdk5/p25 increases total phosphoserine immunoreactivity of TRPA1, consistent with kinase modulation of receptor activity by a phosphorylative mechanism. Moreover, incubation of the TRPA1/Cdk5/p25 transfected HEK293 with roscovitine results in a decrease in phosphoserine immunoreactivity indicating Cdk5 is responsible for increasing phosphoserine phosphorylation. Finally, we observed no phosphoserine immunoreactivity in HEK293 cells that were transfected with the TRPA1 mutant S449A in combination with Cdk5/p25. These data indicate that S449 is the determinant of Cdk5 phosphorylation in the full length TRPA1 channel. We conclude that in addition to T673 serving as a phosphorylation site for Cdk5 regulation of TRPA1 function [19], our current findings indicate that Cdk5 phosphorylation of S449 can also modulate TRPA1 activity.

### 4.6. Conclusions

The mechanism(s) by which Cdk5 affects TRPA1 ion channel activity is an important question to be answered. In the case of modulation of TRPA1 by Cdk5, a ‘phosphoryl only’ mechanism would appear unlikely, as the channel is functional when transfected into cells which lack Cdk5 activity, but is dysfunctional in DRG neurons, in the absence of Cdk5 activity. Thus, Cdk5 influence on TRPA1 channel function (and of TRPV1 function [9]), whether exerted solely via direct phosphorylation or not, appears to require the presence of additional regulatory factors.

A simple model consistent with the present data would be one in which Cdk5 phosphorylation of TRPA1 prevents binding of an allosteric inhibitory factor (a factor significantly expressed in DRG neurons, but not in HEK293 cells) to the receptor. Such an inhibitory factor might be postulated to act by sequestering de-phosphorylated TRPA1 away from the plasma membrane, and/or might help place the plasma membrane-localized ion channel into an inactive/less active conformation or complex. Notably, either of these mechanisms have the potential to be easily reversible – which is consistent with the results observed in the present investigation.

As improved understanding of TRPA1 regulation and functional interactions can potentially inform the development of rationally-designed therapeutics targeting the receptor, the novel findings described in this study have potential utilitarian implications, in addition to enhancing our knowledge of basic nociceptor biology. Further investigations into the mechanistic interactions underlying Cdk5 regulation of TRPA1 (and TRPV1) promise to offer additional insight in this regard.

## Abbreviations

*AITC*: allyl isothiocyanate.
*Cdk5*: Cyclin-dependent kinase 5
*DRG*: dorsal root ganglion
*HEK293*: human embryonic kidney 293
*TRPA1*: Transient receptor potential cation channel, subfamily A, member 1
